# The wearable cardioverter defibrillator as a bridge to reimplantation in patients with ICD or CRT-D-related infections

**DOI:** 10.1186/s13019-017-0669-2

**Published:** 2017-11-25

**Authors:** L. Castro, S. Pecha, M. Linder, J. Vogler, N. Gosau, C. Meyer, S. Willems, H. Reichenspurner, S. Hakmi

**Affiliations:** 1 Department of Cardiovascular Surgery, University Heart Center Hamburg, Hamburg, Germany; 2 Department of Cardiology, Electrophysiology, University Heart Center Hamburg, Hamburg, Germany

**Keywords:** Wearable cardioverter defibrillator, CIED-infection, ICD infection, Sudden cardiac arrest, Lead/ device extraction, Ventricular tachycardia

## Abstract

**Background:**

The approach to treat device infection in patients with implantable cardioverter defibrillator (ICD) or cardiac resynchronization therapy defibrillator (CRT-D) is a challenging procedure. Optimal treatment is complete extraction of the infected device. To protect these patients from sudden cardiac arrest while waiting for reimplantation and to avoid recurrent infection, a wearable cardioverter defibrillator (WCD) seems to be a valuable solution.

Therefore, we investigated the management and outcome of patients with ICD or CRT-D infections using the WCD as a bridge to re-implantation after lead extraction procedures.

**Methods:**

We conducted a retrospective study on consecutive patients who underwent ICD or CRT-D removal due to device-related local or systemic infections. All patients were prescribed a WCD at our center between 01/2012 and 10/2015. All patients returned to our outpatient clinic for regular ICD or CRT-D monitoring initially 1 and 3 months after reimplantation followed by 6-months intervals.

**Results:**

Twenty-one patients (mean age 65.0 ± 8.0 years, male 76.2%) were included in the study. Complete lead extraction was achieved in all patients. While waiting for reimplantation one patient experienced a symptomatic episode of sustained ventricular tachycardia. This episode was converted successfully into sinus rhythm by a single 150 J shock.

Mean follow-up time 392 ± 206 days, showing survival rate of 100% and freedom from reinfection in all patients.

**Conclusion:**

The WCD seems to be a valuable bridging option for patients with ICD or CRT-D infections, showing no recurrent device infection.

## Background

Due to the increasing indications for cardiac resynchronization-therapy devices (CRT-D) and implantable cardioverter defibrillator devices (ICD) for heart failure therapy and prevention of sudden cardiac arrest (SCA), the implantation rates for cardiovascular implantable electronic devices (CIEDs) have increased over time [1, 2]. In this context, there is also a rising number of devices that have to be extracted due to local or systemic device-related infections.

The choice of treatment strategy for the prevention of sudden cardiac arrest (SCA) among patients after removal of an infected implantable cardioverter-defibrillator (ICD) system is still an important clinical challenge. The underlying pathologies – such as, structural heart disease, previous myocardial infarction or genetic abnormality – pose a constant and ongoing risk for ventricular tachycardia. Current guidelines recommend complete removal of the infected device and parenteral antibiotic treatment, followed by two-stage contralateral reimplantation [3]. Care of patients in this period is critical. On one hand, implanting the device too early may result in recurrent infection. On the other hand, patients still remain at risk for SCA.

Using a wearable cardioverter defibrillator (WCD) seems to be a valuable approach to protect patients from SCA while waiting for reimplantation and to avoid too early reimplantation leading to recurrent infection.

We here investigated the management and outcomes of patients with ICD or CRT-D infections using the WCD as a bridge to reimplantation after device extraction procedures.

## Methods

### Patient characteristics

Patient characteristics are shown in Table 1. We conducted a retrospective study on consecutive patients who underwent ICD or CRT-D removal due to local or systemic infection at our center between January 2012 and October 2015. Patient information was de-identified in all cases and all patients were prescribed a WCD (LifeVest, ZOLL, Pittsburgh, PA, USA). We discussed all cases in an interdisciplinary heart team, including a cardiothoracic surgeon and a cardiologist/ electrophysiologist.

**Table 1:** Baseline and clinical characteristics

Patients	( *n* = 21)
Demographics	
Age, years	65 ± 8
Male gender, n (%)	16 (76.2)
Medical history	
Renal failure, n (%)	7 (33.3)
Diabetes mellitus, n (%)	9 (42.85)
Coronary artery disease, n (%)	11 (52.4)
Symptoms	
Pocket infection, n (%)	14 (66.7)
Systemic infection, n (%)	7 (33.3)
Laboratory	
Leucocytes billion/l	8.7 ± 2.2
C-reactive protein mg/l	66.0 ± 61

Continuous variables are expressed as mean ± standard deviation and categorical are expressed by values and percentages

### Removal techniques

All procedures were performed in a hybrid operating room with patients under general anesthesia. For monitoring during lead extraction we used continuous arterial blood pressure measurement via an arterial line placed in the radial artery and 3D transoesophageal echocardiography.

After removal of the generator we did extensive wound debridement and collected specimens from the pocket. Leads were dissected from the scar tissue and the sleeves were removed.

Leads implanted less than 24 months ago were extracted under fluoroscopic guidance with use of a lead locking device (Spectranetics Corporation, Colorado Springs, CO, USA) making the traction procedure more efficient.

When the attempt to remove leads was not successful because of aggressive adhesions, a Spectranetics laser sheath (Spectranetics Corporation, Colorado Springs, CO, USA) was applied in 12 cases. Lead tips were collected for microbiological work up.

### Postoperative management

According to antibiotic sensitivity testing we initiated an antibiotic treatment. All patients were prescribed a WCD and were trained in handling the WCD 1–2 days after explantation. Very thoroughly programming was required due to unavailable antitachycardia pacing in WCD. The recommended programming is 170–220 beats/min for rate detection with VT shock delay programmed from 60 to 180 s. Depending on former device programming and ECG recordings from the explanted device, rate and treatment criteria of the WCD were adjusted and programmed. Although the VT detection can be programmed to as low as 120 beats/min, we avoided to program lower rates than 160 beats/min to prevent inappropriate shocks.

Before discharge appointments were made for every patient to return to our outpatient clinic for first follow-up four weeks after the respective device explantation. A new device was implanted on contralateral side when C-reactive protein (CRP) value and leucocyte counts were within normal ranges and blood culture samples showed negative results.

### Follow-up and endpoints

Patients returned to our outpatient clinic for regular ICD or CRT-D monitoring initially 1 and 3 months after re-implantation followed by 6-months intervals. The primary endpoints of the study were absence of serious arrhythmic complications and freedom from reinfection.

### Definitions

Local infection was defined as the presence of local inflammation-signs (pocket abscess, skin adherence, chronic draining sinus) at the generator pocket or erosion of a lead or device through the skin without clinically evident involvement of the intravenous portion of the lead system [3, 4].

Systemic infection was defined as valvular endocarditis with or without definite involvement of the leads and/or device, occult gram-positive bacteremia (not contaminant) or sepsis. Freedom from reinfection was defined as the absence of such detection during the observational period [3, 4].

Complete procedural success was defined as removal of all infected device elements (generator, leads, sleeves) without any complications or procedure related death.

### Statistical analysis

All data were recorded prospectively into a database and analyzed retrospectively with SPSS statistical software version 23 (IBM SPSS Statistics for Windows, Version 23.0). All continuous variables are expressed as mean ± standard deviation and categorical variables are displayed as numbers and percentages.

## Results

### Patients

Twenty-one patients with a mean age of 65.0 ± 8.0 years were included in the study. The indication for initial device implantation was primary prevention in 10 (47.6%) patients and secondary prevention in 11 (52.4%) patients.

Infection was local in 14 patients (66.7%; 6 ICD; 8 CRT-D) and systemic in 7 patients (33.3%; 4 ICD; 3 CRT-D). In all patients serological infection parameters were elevated, mean leucocytes counts were 8.7 ± 2.2 billion/l (range 4.8–16.2) and mean CRP values were 66 ± 61 mg/dl (range 5–343).

Laser lead extraction had to be performed in 12 (57.1%) patients. Mean procedural time was 74 ± 30 (range 15–150) minutes with a mean fluoroscopic time of 215 ± 164 (range 20–390) seconds. During extraction, one major adverse event occurred. This was a tear at the coronary sinus (CS) during the extraction procedure of a StarFix™ (Medtronic Inc., Minneapolis, MN, USA) active fixation CS lead in a 57-years old male with subsequent need for sternotomy. Under the use of cardiopulmonary bypass the tear was successfully patched and the patient made an uneventful recovery thereafter.

### Microbiology and antibiotic treatment

Antibiotic treatment had been initiated prior to surgery in 8 (38.1%) patients. If necessary, the antibiotic treatment was escalated or adapted following accomplished antibiotic sensitivity testing. After microbiological work up of the pocket specimen and lead tips, *Staphylococcus epidermidis* was the predominant infectious organisms (38.1%) found in swab and on the lead-tips. Mean postoperative duration of antibiotic treatment was 15.0 ± 5.0 (range 7–28) days.

### Reimplantation

When CRP- and leucocyte values were in normal range and 3 blood culture samples showed negative results, a new CIED was successfully implanted on the contralateral side. Before reimplantation we reviewed the initial indication for CIED-implantation and if necessary a different CIED - adapted to the current diagnostic findings - was implanted as show in Table 2. Nine (42.85%) patients were re-implanted with an ICD, 9 (42.85%) patients with CRT-D and 3 (14.3%) patients with subcutaneous-ICD. The mean time to reimplantation was 51.6 ± 16.9 (range 28–108) days.

**Table 2:** Device characteristics

Devices	( *n* = 21)
Indication for implantation	
Ischemic cardiomyopathy, n (%)	7 (33.3)
Non-ischemic cardiomyopathy, n (%)	14 (66.7)
Long QT syndrome, n (%)	1 (4.8)
ARVC, n (%)	2 (9.5)
Secondary prophylaxis, n (%)	11 (52.4)
Explanted	
VVI-ICD, n (%)	5 (23.8)
DDD-ICD, n (%)	6 (28.6)
CRT-D, n (%)	10 (47.6)
Reimplanted	
VVI-ICD, n (%)	6 (28.6)
DDD-ICD, n (%)	3 (14.3)
CRT-D, n (%)	9 (42.8)
S-ICD, n (%)	3 (14.3)
Time to re-implantation, days	51.6 ± 16.9

*ARVC* Arrhythmogenic right ventricular cardiomyopathy

Continuous variables are expressed as mean ± standard deviation and categorical are expressed by values and percentages

### Follow-up

**Fig. 1:**
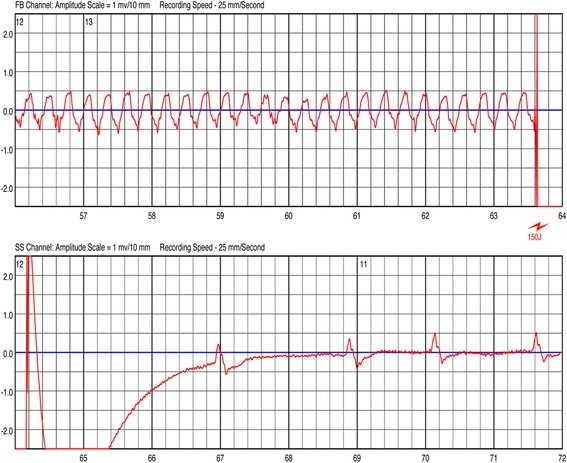
Successful wearable cardioverter defibrillator shock (150 J)

While waiting for reimplantation 1 (4.8%) patient experienced an episode of sustained (> 30 s) ventricular tachycardia 6 weeks after explantation. This episode was converted into sinus rhythm by a single 150 J shock of the WCD (Fig. 1). The patient was admitted to our emergency department for further monitoring and treatment. He did not show any local or systemic infection signs, thus it was decided to implant a new CRT-D.

All patients returned to our outpatient clinic for CIED monitoring after 1 and 3 months and none of the patients showed recurrent infection. The follow up was continued by regular ICD monitoring every 6 months. Mean follow-up time was 454 ± 263 (range 114–1068) days, showing survival rate of 100% and freedom from reinfection in all patients.

All patients were comfortable with wearing WCD and compliance with device use was 100% showing a mean daily wear time of 22.0 (15–24) hours per day.

## Discussion

In this study we have shown that the use of WCD is a safe and efficient solution to protect patients from SCA while waiting for re-implantation of a new ICD/CRT-D device. During the period of WCD treatment, one patient experienced a symptomatic sustained ventricular tachycardia, which has been successfully treated by a single 150 J WCD shock. No inappropriate shocks were delivered. The WCD bridging concept, with the possibility to delay reimplantation until freedom from infection is achieved, enabled for the absence of recurrent infection observed in this study.

For patients with systemic device infection, the complete removal of the device and all leads is a class I recommendation in 2010 AHA/HRS Scientific Statement (AHA) [4]. Conservative treatment of those patients is associated with high mortality and the complete device removal has been shown to significantly reduce morbidity and mortality in those patients.

Furthermore, a two-step approach with an extended period of antibiotic treatment before re-implantation is recommended [4]. In several studies it has been proven that this two-step re-implantation strategy is associated with reduced re-infection rates. The ideal timing for re-implantation is still discussed controversially in literature. However, treatment regimen should be individualized for each patient and clinical situation [4]. In patients with systemic infection and positive blood cultures, antibiotic treatment should be performed for an extended period of time, and blood cultures should be negative for at least 72 h before a re-implantation is conducted [4]. In patients with positive blood cultures and evidence for valvular involvement, implantation of a new transvenous system should even be delayed for at least 14 days, after first negative blood cultures are drawn.

These data underline the importance of a suitable bridging concept after device removal, especially in patients with pacemaker dependency or risk for sudden cardiac arrest after ICD explantation. In patients with pacemaker dependency, as a bridging concept, a temporary RV lead can be implanted, tunneled subcutaneously and connected to an externalized pacemaker device [5].

In ICD/CRT-D patients with device removal due to infection and ongoing risk for SCA, hospitalization of the patient and continuous rhythm monitoring has been the only safe and reasonable treatment option for a long time. However, continuous inpatient monitoring is cost-effective and undesirable to the patient from a quality-of-life standpoint [6]. Furthermore this situation might lead to too early re-implantation of a new ICD/CRT-D device in some cases, carrying a high risk for recurrent infection.

Using a WCD allows extension of the period of antibiotic treatment until re-implantation up to several weeks, if necessary. During this time the patient can be discharged home safely until re-implantation is scheduled [6].

Safety and efficacy of WCD has been proven in clinical studies [7–9]. In a large prospective clinical registry, the WEARIT-II registry, 2000 patients with WCD were included. In this registry, 120 sustained ventricular tachycardia (VT)/ ventricular fibrillation (VF) episodes occurred in 41 patients. Importantly, most of the sustained VTs were not treated by the WCD, while patients used the response button to delay therapy and the VT episodes self-terminated. However, 30 events in 22 patients needed shock delivery due to hemodynamic instability [8]. All patients requiring shock delivery had their VT/VF episode terminated successfully with the first shock, showing the high efficacy of WCD therapy. In the largest WCD registry including 3569 patients, Chung et al. demonstrated a first shock efficacy of 99% [10]. Although a smaller study, our experience of one appropriate and successful shock demonstrated a shock efficacy of 100%.

In contrast to previous published data by Tanawuttiwat et al. and Chung et al. we observed no inappropriate shocks in our study. In the study by Wuttiwat et al. one patient (1.0%) received an inappropriate shock due to oversensitivity of a signal artifact. Regarding inappropriate shocks, across studies with WCDs, the rate is with 0.67–1.4% per month of use, similar to those of ICDs [8, 11, 12]. Reasons that account for inappropriate shocks are noise, supraventricular tachycardia above the preset rate criteria or device malfunction. In contrast to an ICD, the WCD enables the patient to press a response button while awake and abort shock delivery. This tool might help to reduce the rate of inappropriate shock delivery.

Patients being prescribed a WCD need detailed teaching and instructions. On the one hand the patient needs to know all important device functions, like e.g. the response button and on the other hand needs to understand the importance of wearing the WCD to prevent SCA, ensuring a good compliance and high daily wear time.

In our study a mean daily wear time of 22.0 (15–24) hours shows that the WCD is a practical solution, which is easy to handle. Daily wear time, in studies by Tanawuttiwat 2014, Kutyifa et al. was with 20–22.5 h per day comparable. WCD discontinuation rates of up to 22%, which have been described by Feldman et al. were not observed in our series [9]. Another important aspect of the WCD therapy is cost-effectiveness. By avoiding expensive and uncomfortable inpatient monitoring, Healy et al. have shown that use of WCD and discharging patients home is associated with reduced costs and improvement in quality-adjusted-life-year (QALY) analysis, when compared with inpatient monitoring [6].

The protection from SCA with WCD, allowing for an appropriate time of antibiotic therapy with mean duration of 15.0 days was surely one of the reasons for the favourable outcome of our patients, showing no mortality and no recurrence of infection during mean follow-up time of 392 days. Another important factor was the successful lead extraction with complete removal rate of 100%. The combination of these two therapy aspects seems to be the ideal treatment option in patients with ICD/CRT device infection.

## Conclusion

The WCD is a safe, comfortable and cost-effective bridging solution for these patients, showing survival rate of 100% and no recurrent device infection after a mean follow-up time of 454 ± 263 (range 114–1068) days.

## Limitations

The major limitation of this study is the small number of patients and the retrospective design with its inherent limitations and possible bias. In the future, larger prospective studies are needed to investigate safety and efficiency of the wearable cardioverter defibrillator as a bridge to reimplantation solution.

## Abbreviations

CIED: Cardiovascular Implantable Electronic Device
CRT: Cardiac Resynchronization Therapy
ICD: Implantable Cardioverter Defibrillator
SCA: Sudden Cardiac Arrest
VF: Ventricular Fibrillation
VT: Ventricular Tachycardia
WCD: Wearable Cardioverter Defibrillator
